# Supplementary material for: Modulation of Cellular MicroRNA by HIV-1 in Burkitt Lymphoma Cells—A Pathway to Promoting Oncogenesis
Source: Genes (Basel). 2021 Aug 24;12(9):1302. doi: 10.3390/genes12091302 (PMC8468732; doi:10.3390/genes12091302)
Supplement: Supplementary file 1 [file genes-12-01302-s001.zip › genes-1318761-supplementary.pdf]

Supplementary Table 1.1: Summary of miRNAs reported to be deregulated in Diffuse Large B-cell lymphoma

Subtype	Upregulated miRNAs	Downregulated miRNAs	Reference
Germinal Centre B-cell like	miR-10398-3p, NOVEL00260M, NOVELM00010M, miR-10b-5p, miR-423-3p, miR-301a-5p, miR-598, miR-181a-5p, miR-30e-3p, miR-744-5p, miR-4746-5p, miR-1270, miR-3074-5p, miR-589-3p, miR-151a-3p, miR-331-3p, miR-3934-3p, miR-589-5p, miR-210, miR-138-1-3p, miR-28-5p, miR-339-3p, miR-196b-5p, miR-151b, miR-129-2-3p, miR-664a-3p, miR-28-3p, miR-582-3p, miR-3681-5p, miR-129-1-3p, miR-582-5p, miR-138-5p, miR-129-5p, miR-3150b-3p	*	Lim *et al.,* 2015
	*	miR-28	Schneider *et al.,* 2014
	miR-18a	*	Alencar *et al.,* 2011
	miR-17, miR-18a, miR-19a, miR-20a, miR-19b, miR-92a	*	Fassina *et al.,* 2012
	miR-17-5p, miR-150, miR-145, miR-328	*	Roehle *et al.,* 2008
	miR-17/92, miR-181a, miR-221	*	Lawrie *et al.,* 2008
	miR-17, miR-19b, miR-20a	*	Culpin *et al.,* 2010
	miR-28-3p, miR-28-5p, miR-129-3p, miR-589, miR-331-5p, miR-597	*	Iqbal *et al.,* 2015
Non-Germinal Centre B-cell like	miR-10397-5p, NOVELM00288M, miR-155-3p, miR-222-5p, miR-148a-5p, miR-222-3p, miR-625-3p, miR-363-3p, miR-30d-3p, miR-30b-3p, miR-221-3p, miR-92a-1-5p, miR-21-3p, miR155-5p, miR625-5p, miR-29b-1-5p, miR-20a-5p, miR-17-5p, miR-106a-5p, miR-503-5p, miR-424-5p, miR-302a, miR-10b-5p	*	Lim *et al.,* 2015
	miR-181, miR-222	*	Alencar *et al.,* 2011
	*	miR-34a	Craig *et al.,* 2012
	miR-155, miR-29b, miR-146a, miR-365, miR-30b, miR-26b, miR-374, let-7f, miR-9, miR-9-3p, miR-34b	*	Di Lisio *et al.,* 2012
	miR-23a, miR-24-2, miR-27a	*	Kong *et al.,* 2010
	miR-150, miR-145, miR-328	*	Roehle *et al.,* 2008
	miR-155, miR-21, miR-210, miR-221	*	Lenz *et al.,* 2008
	miR-155, miR-21, miR-22, miR-146a, miR-146b, miR-500, miR-363, miR-574-3p, miR-547-5p, miR-222	*	Malumbres *et al.,* 2009
	miR-29a, miR-92a, miR-106a, miR-720, miR-1260, miR-1280	*	Culpin *et al.,* 2010
	miR-155, miR-542-3p	*	Iqbal *et al.,* 2015

* no miRNAs were reported as downregulated/upregulated in this study (miR-microRNA)

Table 1.2: Summary of miRNAs reported to be deregulated in Burkitt lymphoma

Upregulated miRNAs	Downregulated miRNAs	Reference
miR-371, miR-185, miR-93, miR-326, miR-339, miR-485, miR-193a, miR-448, miR-202, miR-483, miR-26a, miR-328, miR-192, miR-429, miR-324, miR-340, miR-105, miR-124	miR-221, miR-30a, miR-146a, miR-146b, miR-26b, miR-23a, miR-30d, miR-107, miR-103, miR-222, miR-26a, miR-30a, miR-142, miR-23b, miR-342, miR-29b, miR-34b, miR-9	Lenze *et al.,* 2011
miR-19a, miR-18a, miR-19b, miR-20a, miR-17-3p, miR-17-5p, miR-92, miR-106a, miR-130b, miR-128b, miR-7, miR-206, miR-370, miR-494, miR-148a, miR- 20b	miR- 210, let-7e, miR-215, miR-144, miR-451, miR-101, miR-125b, miR-139, miR-140, miR-142-3p, miR-146a, miR15a, miR-150, miR-16, miR-195, miR-22, miR-223, miR-23a, miR-23b, miR-24, miR-26a, miR-26b, miR-29a, miR-29b, miR-29c, miR-30c, miR-30e-3p, miR-30e-5p, miR-34a, miR-99b, let-7a, miR-155, miR-196a, miR-342	Robertus *et al.,* 2010
miR-17-5p, miR-20a, miR-9-3p	*	Onnis *et al.,* 2010
miR-210, miR-494, miR-575, miR-202, miR-801, miR-370, miR-765, miR-188, miR-296, miR-560, miR-663, miR-181, miR-574, miR-197, miR-92, miR-20b, miR-20a, miR-17-5p, miR-106, miR-93, miR- 422, miR-17-3p, miR-18a, miR-130, miR-19b, miR-19a	miR-29b, let-7d, miR-155, miR-15a, let-7e, miR-23b, miR-768-5p, miR-148a, miR-331, miR-27b, miR-28, miR-363, miR-98, let-7a, let-7f, let-7g, miR-29a	Bueno *et al.,* 2011

* no miRNAs were reported as downregulated in this study
miR - microRNA

Table 1.3. MiRNAs (n = 188; 192a format) for microarray profiling, spotted in duplicate, including four controls.

let-7a-2-3p	let-7a-1-3p	let-7a-5p	let-7b-5p	let-7b-3p	let-7c-5p	let-7d-5p	let-7e-5p	let-7e-3p	let-7f-5p	U6	let-7f-1-3p	let-7g-5p	let-7i-5p	miR-100-5p	miR-100-3p	miR-101-3p	miR-103a-3p	miR-105-5p	miR-105-3p	miR-106a-5p	miR-106a-3p	miR-106b-5p	miR-106b-3p
let-7a-2-3p	let-7a-1-3p	let-7a-5p	let-7b-5p	let-7b-3p	let-7c-5p	let-7d-5p	let-7e-5p	let-7e-3p	let-7f-5p	U6	let-7f-1-3p	let-7g-5p	let-7i-5p	miR-100-5p	miR-100-3p	miR-101-3p	miR-103a-3p	miR-105-5p	miR-105-3p	miR-106a-5p	miR-106a-3p	miR-106b-5p	miR-106b-3p
miR-107	miR-124-5p	miR-125b-5p	miR-126-3p	miR-126 -5p	miR-127-3p	miR-130b-3p	miR-132-3p	miR-133a-3p	miR-138-5p	miR-140-3p	miR-141-3p	miR-142-3p	U44	miR-143-3p	miR-143-5p	miR-145-3p	miR-145-5p	miR-146a-3p	miR-146b-3p	miR-146b-5p	miR-148a-3p	miR-148a-5p	miR-149-5p
miR-107	miR-124-5p	miR-125b-5p	miR-126-3p	miR-126-5p	miR-127-3p	miR-130b-3p	miR-132-3p	miR-133a-3p	miR-138-5p	miR-140-3p	miR-141-3p	miR-142-3p	U44	miR-143-3p	miR-143-5p	miR-145-3p	miR-145-5p	miR-146a-3p	miR-146b-3p	miR-146b-5p	miR-148a-3p	miR-148a-5p	miR-149-5p
miR-150-5p	miR-150-3p	miR-151a-3p	miR-155-5p	miR-155-3p	miR-15a-3p	miR-15b-5p	U48	miR-17-5p	miR-17-3p	miR-18a-5p	miR-18b-5p	miR-185-5p	miR-188-5p	miR-191-5p	miR-192-5p	miR-193a-5p	miR-194-5p	miR-194-3p	miR-195-5p	miR-195-3p	miR-196a-5p	miR-196a-3p	miR-196b-5p
miR-150-5p	miR-150-3p	miR-151a-3p	miR-155-5p	miR-155-3p	miR-15a-3p	miR-15b-5p	U48	miR-17-5p	miR-17-3p	miR-18a-5p	miR-18b-5p	miR-185-5p	miR-188-5p	miR-191-5p	miR-192-5p	miR-193a-5p	miR-194-5p	miR-194-3p	miR-195-5p	miR-195-3p	miR-196a-5p	miR-196a-3p	miR-196b-5p
miR-197	miR-199a-3p	miR-19a-3p	miR-19b-3p	U6	miR-20b-5p	miR-200c-3p	miR-202-5p	miR-205-5p	miR-21-5p	miR-210-3p	miR-214-3p	miR-215-5p	miR-218-5p	miR-22-3p	miR-221-3p	miR-222-3p	miR-223-5p	miR-224-5p	miR-23a-3p	miR-23b-3p	miR-24-3p	miR-25-3p	miR-26a-5p
miR-197	miR-199a-3p	miR-19a-3p	miR-19b-3p	U6	miR-20b-5p	miR-200c-3p	miR-202-5p	miR-205-5p	miR-21-5p	miR-210-3p	miR-214-3p	miR-215-5p	miR-218-5p	miR-22-3p	miR-221-3p	miR-222-3p	miR-223-5p	miR-224-5p	miR-23a-3p	miR-23b-3p	miR-24-3p	miR-25-3p	miR-26a-5p
miR-26a-1-3p	miR-26b-5p	miR-26b-3p	miR-27a-3p	miR-27b-3p	miR-28-3p	miR-29a-3p	miR-29b-3p	miR-29c-3p	miR-296-3p	miR-30a-3p	miR-30a-5p	miR-30b-3p	miR-30c-5p	miR-30d-5p	miR-30e-3p	U44	miR-301a-3p	miR-31-5p	miR-32-5p	miR-320a	miR-324-3p	miR-324-5p	miR-326
miR-26a-1-3p	miR-26b-5p	miR-26b-3p	miR-27a-3p	miR-27b-3p	miR-28-3p	miR-29a-3p	miR-29b-3p	miR-29c-3p	miR-296-3p	miR-30a-3p	miR-30a-5p	miR-30b-3p	miR-30c-5p	miR-30d-5p	miR-30e-3p	U44	miR-301a-3p	miR-31-5p	miR-32-5p	miR-320a	miR-324-3p	miR-324-5p	miR-326
miR-328-3p	miR-331-3p	miR-339-3p	miR-339-5p	miR-340-5p	miR-342-5p	miR-342-3p	miR-345-5p	miR-34a-5p	miR-34b-5p	miR-361-5p	miR-363-3p	miR-365a-3p	miR-370-3p	miR-374a-5p	miR-422a	miR-423-5p	miR-425-5p	miR-429	miR-448	miR-454-3p	miR-455-3p	miR-483-3p	miR-484-3p
miR-328-3p	miR-331-3p	miR-339-3p	miR-339-5p	miR-340-5p	miR-342-5p	miR-342-3p	miR-345-5p	miR-34a-5p	miR-34b-5p	miR-361-5p	miR-363-3p	miR-365a-3p	miR-370-3p	miR-374a-5p	miR-422a	miR-423-5p	miR-425-5p	miR-429	miR-448	miR-454-3p	miR-455-3p	miR-483-3p	miR-484-3p
miR-485-3p	U48	miR-494-3p	miR-497-5p	miR-513a-3p	miR-516b-3p	miR-520d-3p	miR-520d-5p	miR-520f-5p	miR-520f-3p	miR-532-5p	miR-563	miR-573	miR-574-3p	miR-575	miR-582-3p	miR-582-5p	miR-590-3p	miR-595	miR-624-5p	miR-624-3p	miR-627-3p	miR-627-5p	miR-628-5p
miR-485-3p	U48	miR-494-3p	miR-497-5p	miR-513a-3p	miR-516b-3p	miR-520d-3p	miR-520d-5p	miR-520f-5p	miR-520f-3p	miR-532-5p	miR-563	miR-573	miR-574-3p	miR-575	miR-582-3p	miR-582-5p	miR-590-3p	miR-595	miR-624-5p	miR-624-3p	miR-627-3p	miR-627-5p	miR-628-5p
miR-629-5p	miR-634	miR-650	miR-660-5p	miR-7-2-3p	miR-765	miR-766-3p	miR-769-5p	miR-9-3p	miR-9-5p	miR-92a-3p	miR-92a-1-5p	miR-92a-2-5p	miR-93-3p	miR-95-3p	miR-96-3p	miR-98-5p	miR-99a-5p	miR-16-5p	miR-142-5p	miR-20a-5p	miR-30e-3p	miR-486-5p	BLANK
miR-629-5p	miR-634	miR-650	miR-660-5p	miR-7-2-3p	miR-765	miR-766-3p	miR-769-5p	miR-9-3p	miR-9-5p	miR-92a-3p	miR-92a-1-5p	miR-92a-2-5p	miR-93-3p	miR-95-3p	miR-96-3p	miR-98-5p	miR-99a-5p	miR-16-5p	miR-142-5p	miR-20a-5p	miR-30e-3p	miR-486-5p	BLANK

POSITIVE CONTROLS | NEGATIVE CONTROL | UPREGULATED | DOWNREGULATED | BOTH UP & DOWN | NO RESULT*

*No reported differential expression in B-cell lymphomas and/or cancer (added onto array for miRNA family involvement)

References: See Bueno *et al.,* (2011); Chang *et al.,* (2008); Chiang *et al.,* (2013); Di Lisio *et al.,* (2012); Forte *et al.,* (2012); Imig *et al.*, (2010); Jima *et al.,* (2010); Leucci *et al.,* (2008); Linda *et al.,* (2010); Malumbres *et al.,* (2009); Robertus *et al.,* (2010); Sampson *et al.,* (2007) (Appendix B)
